# The Prevalence of Potentially Inappropriate Prescribing in Two Family Health Units in Portugal

**DOI:** 10.7759/cureus.49617

**Published:** 2023-11-29

**Authors:** Vera C Fialho, Rita Cardoso, Sofia Fernandes

**Affiliations:** 1 Family Medicine, Unidade de Saúde Familiar (USF) Novo Mirante, Agrupamento de Centros de Saúde Loures e Odivelas (ACES Loures - Odivelas), Odivelas, PRT; 2 Family Medicine, Unidade de Saúde Familiar (USF) Magnólia, Agrupamento de Centros de Saúde Loures e Odivelas (ACES Loures - Odivelas), Odivelas, PRT

**Keywords:** family medicine, deprescribing, frail, elder, potentially inappropriate prescribing

## Abstract

Objective: Polypharmacy and potentially inappropriate prescribing (PIP) are growing concerns in the ageing population. They carry the risk of increasing adverse effects, medical interactions, and difficulties managing the medication. Few studies in Portugal evaluate the prevalence of polypharmacy and PIP in primary care. No previous studies in the primary care setting in Portugal have been conducted using the European Union (EU)(7)-PIM (potentially inappropriate medication) list tool. In this study, we aimed to estimate the prevalence of polypharmacy and PIP in two family health units in Portugal.

Methods: To answer this question, we enrolled a sample of 361 elderly patients from two family health units in a descriptive observational transversal study. We randomly selected patients, consulted their prescription records in the previous 12 months, and applied the EU(7)-PIM list tool, validated for the Portuguese population. The data was then analyzed using descriptive and inferential statistics and the Statistical Package for the Social Sciences (IBM SPSS Statistics for Windows, IBM Corp., Version 24.0, Armonk, NY).

Results: Our results showed a prevalence of 79.8% of polypharmacy in the elderly population and 73.4% of PIP. These values are higher than predicted in the literature, but different screening tools have been used among papers. The mean number of prescribed drugs per patient was nine in one unit and seven in the other, and the mode was eleven per patient. The most identified PIP-associated drugs were proton pump inhibitors in 46.4% of the patients in one unit and 43.7% in the other. We also found a statistically significant higher prevalence of PIP and polypharmacy in females and patients over 75 years.

Conclusion: From a prevalence perspective, we found higher-than-expected prevalences of PIP and polypharmacy in our population. Contributing factors might be a higher ageing index in the Portuguese population, modern practices using combination therapy, and the use of a screening tool that does not take into account the personal clinical history of patients. Further limitations involve only including patients with follow-up in the units studied. Even so, it suggests both PIP and polypharmacy as concerns to address, and we will strive to educate both health teams on PIP, polypharmacy, and deprescribing. We also emphasize the need to widen the study to other family health units.

## Introduction

The protocol for this article was previously displayed as a poster at the WONCA 2023 Europe meeting in Brussels on June 7, 2023. Results were presented as an oral communication at the 43º Congresso Português de Geriatria e Gerontologia on November 16, 2023.

Ageing in the Portuguese population

The increase in life expectancy and the progressive reduction in the birth rate have led through the last decades to population ageing in developed countries. Portugal is no exception to this tendency [1,2]. In 2020, 22.3% of the Portuguese population was over 65 years old, in contrast to only 9.7% in 1971 [3]. The current ageing index in Portugal is 182.7%, with a total age dependency ratio of 57.1% [4].

This ageing population leads to the need to adequate our medical practice to an age group with its challenges; medical prescription is among the most pressing [5-7]. Some of the difficulties posed by the elderly population are polypharmacy, potentially inappropriate prescribing, and deprescribing [7,8].

Polypharmacy

Polypharmacy is the regular usage of at least five to ten drugs, and its prevalence is between 20% and 60% in the Portuguese population [7,9,10]. It depends on factors such as age, patient comorbidities, follow-up with more than one doctor simultaneously, use of out-of-date clinical records, and absence of regular surveillance of medical prescriptions [11,12].

It carries the risk of pharmacological interactions, adverse effects, and hospital admission. In the elderly, it is especially relevant since they are more vulnerable to polypharmacy due to increased comorbidities [13,14]. The elders also have risk adverse effects due to the pharmacodynamic changes caused by ageing and iatrogenesis and face difficulties managing complex therapeutic regimens [15,16]. Polypharmacy, however, does not necessarily mean inappropriate prescribing [17].

Potentially inappropriate prescribing

Potentially inappropriate prescribing (PIP) consists of the use of medication which in a particular patient might carry more risk than benefit [18]. Its identification is a fundamental first step towards deprescribing and control of inappropriate polypharmacy [19]. In order to identify PIP, several tools might be used, such as Beers criteria, STOPP/START, IPET Tool, MAI Index, ACOVE criteria, and the European Union (EU)(7)-PIM (potentially inappropriate medication) list [19-21].

There is yet to be a consensus on which is the better tool to screen PIP, and the choice depends on application context (day-to-day practice or investigation), available patient information, available time to apply the criteria, and even the user's proficiency [22].

In this study, the authors opted for the EU(7)-PIM list for its simplicity and ease of use since it is a shorter list that does not need access to a deep knowledge of the patient clinical record; for its specificity as a screening tool in elderly populations and for its validity and operationalization in the Portuguese population [9,23,24].

PIP in Portugal: Current knowledge

In Portugal, there have been some studies dedicated to PIP [5,9,17]. In 2019, Urzal studied the prevalence of PIP in a hospital ward, which was between 11.2% and 17%, using deprescribing guidelines from the Bruyère Research Institute [17].

In 2021, Reis-Pina studied PIP in a palliative setting. The mean age was 78 years, and the prevalence of PIP was 90.5%, mainly due to the prescription of proton pump inhibitors and therapeutic obstinacy. This study used the STOPP-Frail (Screening Tool of Older Persons' Prescriptions in Frail adults with a limited life expectancy) criteria [18].

In the primary care setting, Castilho studied in 2021 the prevalence of PIP in the patients of a family health unit. They used the Beers criteria and estimated a 62.05% prevalence of polypharmacy and a 40.7% prevalence of PIP, mainly due to the prescription of proton pump inhibitors and benzodiazepines [25].

Objectives 

The main objective of this study is to estimate the prevalence of PIP and polypharmacy (≥5 drugs) in the elderly population of two family health units in Portugal in the primary care setting. Other objectives included identifying the most commonly PIP-associated drug and evaluating whether there were differences in PIP among both units, gender, and age groups.

With this work, the authors expected to provide knowledge regarding the prevalence of PIP in Portugal and identify potential targets for future education on prescribing.

## Materials and methods

We conducted this descriptive observational transversal study between April and June 2023 after submission to the local ethical committee. This study took place in two family health units on the outskirts of Lisbon: Unidade de Saúde Familiar Novo Mirante (USFNM) and Unidade de Saúde Familiar Magnólia (USFM). Patients were recruited in April of 2023. Data was collected between May and June 2023.

Population

The target population was elderly patients (≥ 65 years) of the two family health units signed up in this study. Inclusion criteria were patients enrolled in both family health units with appointments in the last year either in person or by phone.

Exclusion criteria included a death record at the time of criteria application, patients with no appointments at the units in the last year, or patients with no prescription at the units for at least one year before the criteria application.

Data sources

Two national platforms were used to collect data. MIM@UF®, a restricted access platform available only in the primary health care intranet, provided patients’ demographic data: national health number and family health unit [26].

In order to obtain prescription data, the national electronic prescription platform PEM® was consulted [27]. PEM® is a restricted access platform available only to registered prescribing physicians. In this platform, we consulted drugs prescribed in the last 12 months, with which we applied the EU(7)-PIM list to obtain the PIP-associated drugs.

Variables

These included both numeric and categoric variables, which are listed in Table 1.

**Table 1:** Variables present in this work by type and outcome PIP: potentially inappropriate prescribing, EU(7)-PIM: European Union (7)-Potentially Inappropriate Medication list, USF: Unidade de Saúde Familiar

Variable	Type	Outcome
Age	Numeric and discrete	Measured in years and calculated from birth year in the PEM® platform at the date of sample data collection
Gender	Categorical and dichotomous	Male or female, obtained from the PEM® platform
Family health unit of origin	Categorical and dichotomous	USF Novo Mirante or USF Magnólia, obtained from the MIM@UF® platform
Number of places of prescription	Numeric and discrete	Measured from counting in the PEM® platform the prescription places where prescribing took place for each patient in the 12-month interval
Number of drugs prescribed	Numeric and discrete	Measured from counting prescribed drugs in the 12-month interval on the PEM® platform
Number of PIP drugs prescribed	Numeric and discrete	Measured from counting PIP-associated drugs prescribed in the 12-month interval on the PEM® platform and after application of the EU(7)-PIM list
Class of PIP-associated drug prescribed	Categorical	Measured as defined in the EU(7)-PIM list, and noted as the class or drug defined as PIP by the list, which includes 184 different drugs or therapeutic classes [5]

Bias

Patients were randomly selected to address selection bias, and the sample was calculated to be representative. We consulted with a data analyst to address bias in data analysis and interpretation.

Sample size

Total patients for each unit, listed by national health number, were consulted on the local demographic database platform, MIM@UF. We stratified patients into four subgroups: females aged 65-74, females aged 75 and over, males aged 65-74, and males aged 75 and over. A total of 5955 eligible patients were noted.

We calculated a population sample for a confidence interval of 95% (Z-score of 1.96) with an error margin of 5%. We used the modified Cochran formula for finite populations to calculate the sample size [28]. For each subgroup, the sample was calculated using the formula, and the results are shown in Table 2.

**Table 2:** Distribution of population and sample values calculated by each subgroup USFNM: Unidade de Saúde Familiar Novo Mirante; USFM: Unidade de Saúde Familiar Magnólia

		USFNM - population	USF NM - sample	USFM - population	USFM - sample
65-74 years	Male	764	46	481	29
	Female	910	55	586	36
≥75 years old	Male	800	49	491	30
	Female	1225	74	698	42
Total		3699	224	2256	137

We then randomized patients from the eligible population for each subgroup until we reached the sample size. After we applied exclusion criteria, further patients from the previous randomization were selected as needed.

Data collection

We used the patient’s national health number to access the PEM® platform, where we obtained the patient’s birth date, gender, number of prescribed drugs in the last 12 months, and the drug list from where we could apply the EU(7)-PIM list.

We registered on our database age, gender, family health unit of origin, number of prescribed drugs, number of PIP-associated drugs identified, and their name or pharmacological class. No further personal data from the patients was collected. All data was registered in a password-protected offline database to increase security.

Statistical analysis

We consulted with a data analyst and used SPSS® version 24 (Statistical Packages for Social Sciences, IBM Corp., Armonk, NY) to analyse the data using descriptive and inferential statistics. Categorical data were presented using numbers, percentages, means, modes, and standard deviation. For the inferential analysis, we applied Kolmogorov-Smirnov as a normality test. We then used nonparametric tests such as the Mann-Whitney test to evaluate and compare the distribution of the ordinal variables such as the number of PIP-associated drugs and age. We used the Chi-Square test to verify the association between the family health units and the presence of PIP.

## Results

**Figure 1:**
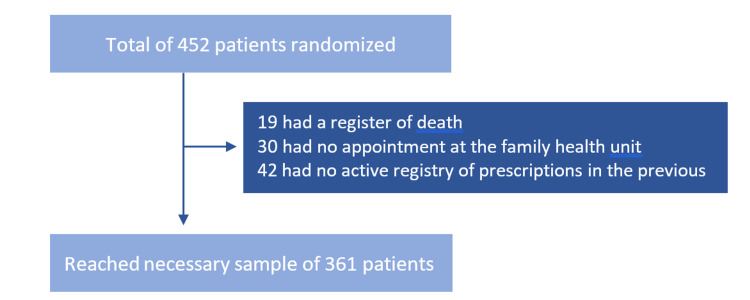
Flowchart of patients sampled and excluded from this study after applying exclusion criteria

To reach the calculated sample of 361 patients, we had to analyse 452 patients’ prescription records from both health units. We excluded 91 patients after applying the exclusion criteria: 19 had a register of death, 30 had no appointment at their family health unit in the previous two years, and 42 had no active registry of prescriptions in the previous year, as shown in Figure 1.

**Figure 2:**
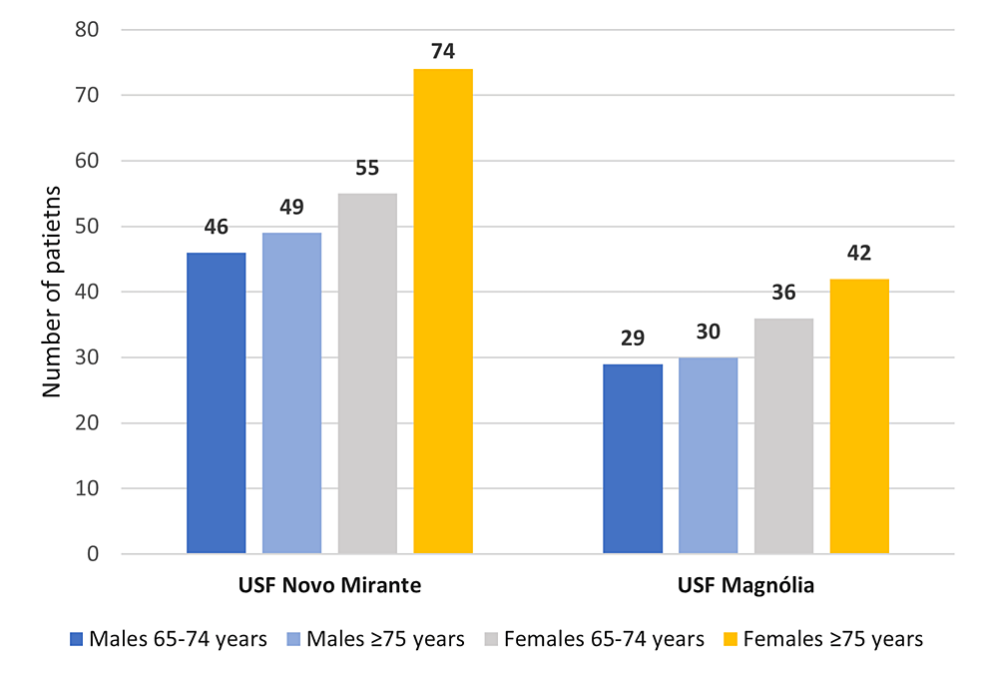
Total number of patients per family health unit and age group after applying the exclusion criteria USF: Unidade de Saúde Familiar

From the sample of 361 patients, 62% (n=224) were from USFNM, and 38% (n=137) belonged to USFM, as shown in Figure 2.

Patients enrolled were aged 65 to 98 years, with a mean of 76.7 years and a standard deviation of 7.9 years. Most were in the age subgroup of over 75 years old (54.2%), and 45.8% in the 65-74 years subgroup. A total of 55.4% of the patients were female (n=200), and 44.6% were male (n=161). The Kolmogorov-Smirnov test showed that the sample did not follow a normal distribution.

No missing data was recorded throughout this study. Our sample was random, representative of the age subgroups of the population of each family health unit, and its size respected the confidence interval of 95%.

Polypharmacy

The prevalence of polypharmacy was 79.8% (n=288): 83.9% (n=188) at USFNM and 72.9% (n=100) at USFM.

**Figure 3:**
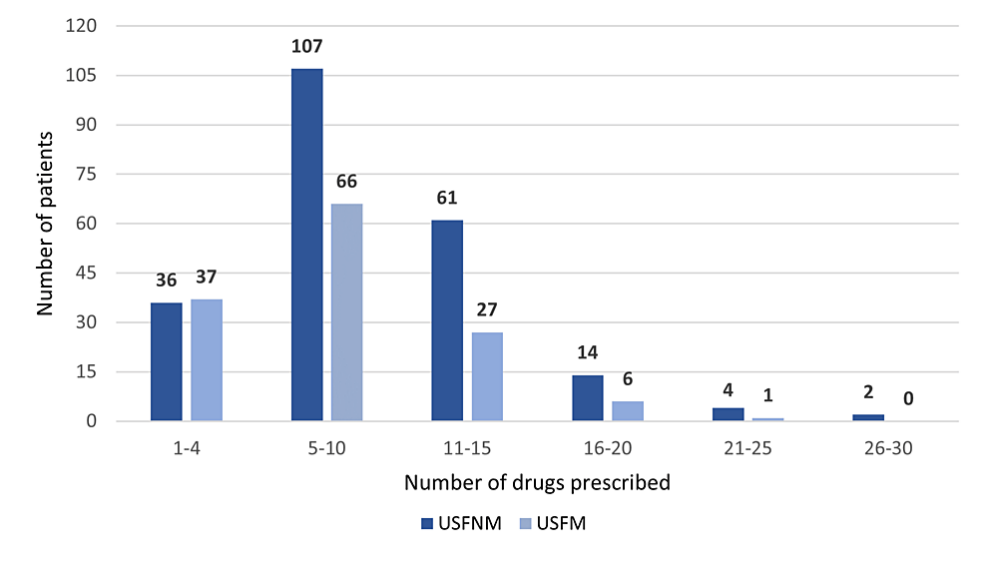
Number of drugs prescribed per patient in each family health unit USFNM: Unidade de Saúde Familiar Novo Mirante; USFM: Unidade de Saúde Familiar Magnólia

In USFNM, the mean number of prescribed drugs per patient was nine, while in USFM, it was seven, with a statistically significant difference (p=0.003). The mode was eleven and eight drugs, respectively. The number of total prescribed drugs per patient in the 12-month period analysed is shown per family health unit in Figure 3.

Of the 288 patients with polypharmacy, 64.9% (n=187) were only prescribed drugs at their respective family units; 35.1% (n=101) had more than one simultaneous prescribing location. A total of 5.5% of the patients (n=16) had three or more simultaneous prescribing doctors, including their primary care doctor, and hospital and private clinic doctors. The number of prescription sites, although different between units, did not impact the difference in polypharmacy and PIP between family units.

Potentially inappropriate prescribing

**Figure 4:**
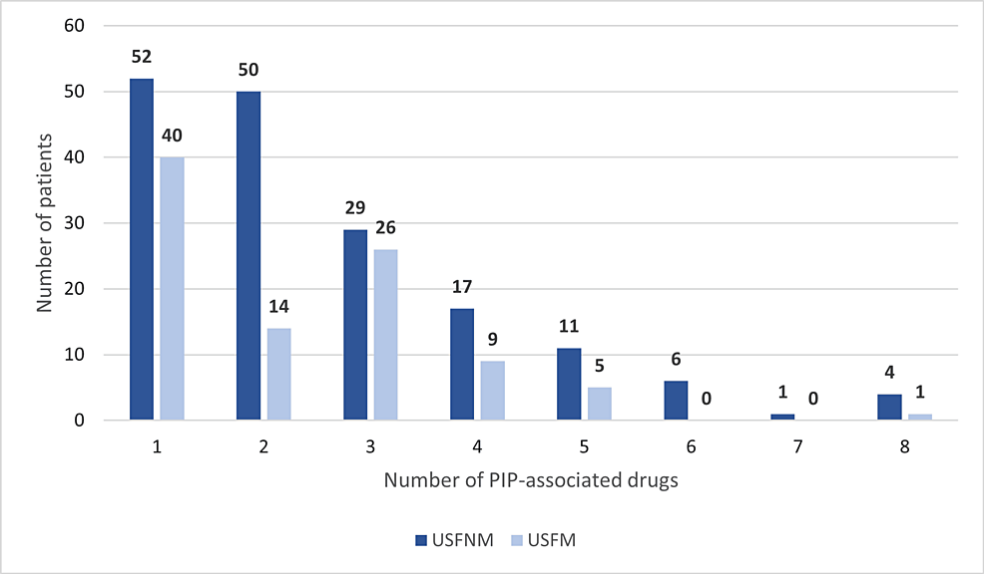
Number of PIP-associated drugs prescribed per patient in each family health unit USFNM: Unidade de Saúde Familiar Novo Mirante; USFM: Unidade de Saúde Familiar Magnólia, PIP: potentially inappropriate prescribing

The prevalence of PIP was 73.4% (n=265): 75.9% (n=170) at USFNM and 69.3% (n=95) at USFM. Of the patients associated with PIP, 62% (n=164) were only prescribed drugs at their respective family units. PIP-associated drugs per patient varied between one and eight. The number of PIP-associated drugs per patient is shown in Figure 4.

There was no statistically significant association between the unit and the presence of PIP (p=0.177). However, there was a statistically significant difference in the number of PIP-associated drugs per patient between both units, with 2.57 drugs/patient in USFNM compared to 1.57/patient in USFM (p=0.000), suggesting that the variables are independent.

The most common PIP-associated drugs identified were proton pump inhibitors (43.7%): 46.4% in USFNM, and 38.7% in USFM. Other PIP-associated drugs frequently identified in USFNM were tramadol, alprazolam, bromazepam, and apixaban. In USFM we identified frequent use of tramadol, alprazolam, apixaban, naproxen, and cyclobenzaprine. In Table 3, we list the 10 most prevalent PIP-associated drugs in each family health unit and their prevalence.

**Table 3:** Ten most prevalent PIP-associated drugs by family health unit; their prevalence in absolute number and percentage USFNM: Unidade de Saúde Familiar Novo Mirante; USFM: Unidade de Saúde Familiar Magnólia, PIP: potentially inappropriate prescribing

PIP-associated drugs in USFNM			PIP-associated drugs in USFM		
Proton pump inhibitors	n=104	46.4%	Proton pump inhibitors	n=53	38.7%
Tramadol	n=28	12.5%	Tramadol	n=19	13.8%
Alprazolam	n=18	8%	Alprazolam	n=16	11.7%
Bromazepam	n=13	5.8%	Apixaban	n=8	5.8%
Apixaban	n=12	5.4%	Naproxen	n=8	5.8%
Naproxen	n=12	5.4%	Cyclobenzaprine	n=7	5.1%
Iron	n=11	4.9%	Zolpidem	n=6	4.4%
Sitagliptin	n=11	4.9%	Bromazepam	n=6	4.4%
Zolpidem	n=10	4.5%	Paroxetine	n=5	3.6%
Cyclobenzaprine	n=10	4.5%	Lorazepam	n=5	2.2%

There was also a statistically significant difference in the number of PIP-associated drugs per gender and age subgroup, with a more significant prevalence in females and patients over 75 years (p= 0.001).

## Discussion

There are no previous studies published that evaluate the prevalence of PIP using the EU(7)-PIM list in the Portuguese primary care setting, and no works so far study the prevalence of PIP in the Lisbon area.

The prevalence of polypharmacy was higher in our population than in other Portuguese works in the literature [8,25]. Globally, polypharmacy was 79.8% of our population. However, the definition of how many drugs constitute polypharmacy varies in the literature. So, studies considering a higher cut-off may have lower estimates, which makes the comparison of results harder.

Contributing factors to polypharmacy in our population might be the ageing of the population and better control of chronic disease, leading to a higher life expectancy with more comorbidities [7,13,14]. This idea is supported by the statistically significant higher prevalence in patients over 75 and females, who usually have a higher life expectancy. Also, there is the crescent use of combination therapy rather than monotherapy in common diseases such as diabetes [29]. Another contributing factor to the prevalence of polypharmacy is the number of prescription places per patient, found in this study to be more than one in 35.1% of patients [11,12].

The prevalence of PIP-associated drugs was also higher in our population than in other works in the literature [17,25]. Globally, we estimated a prevalence of 73.4% of PIP in our population. However, since most other works have used different screening tools, it is challenging to compare results.

Contributing factors might be the usage of the EU(7)-PIM list, a tool that does not consider the patient's clinical record, only the medication prescribed. As such, medications otherwise indicated might be considered inappropriate, taking only into account the patient's elderly status. As an example, one frequent PIP-associated drug was apixaban; however, an elderly patient might benefit from this class in the event of atrial fibrillation [30].

As for the most frequent PIP-associated drug identified, we would like to highlight the proton pump inhibitors, with a prevalence of 43.7% in both family health units. The authors could not ascertain the clinical indications for their prescription since, to do that, we would need access to clinical records. However, the authors believe these results align with the widespread use of this class of drugs in Portugal, as shown in the work of Reis-Pina et al. in 2021 [7].

Comparing the prevalence of polypharmacy and PIP between the two family health units, we found no statistically significant differences. These findings hint at a broader population-based problem, transversal to both settings. However, the sample of this work was small, and more extensive studies are needed.

As in polypharmacy, there was a statistically significant difference in the number of PIP-associated drugs, with a higher prevalence in females and patients over 75 years. Contributing factors might be, again, the higher life expectancy and more comorbidities [6,8].

Concerning the limitations of this study, the authors would like to point out the tool used for screening, which is easy to apply and allows the comparison of results between countries but loses specificity by not taking the patient clinical record into account; another limitation is the small size of the population and sample.

Other limitations include excluding patients who did not have a recent follow-up in one of the family health units, which may exclude patients in nursing facilities or other admission settings. As noted in some works, these patients might receive medical attention in other prescription settings and might be at risk of polypharmacy and PIP [17,18].

Since it was not possible to access the clinical records of the patient, and this study was based on prescription records alone, it was not possible to ascertain drugs that might have been suspended or switched during the analysed period. This limitation may contribute to drugs that might have already been suspended being counted towards the total number of drugs per patient, thus overestimating the prevalence of polypharmacy.

In the future, the authors would like to widen this study to other family health units in the primary care setting in Portugal.

## Conclusions

With this work, we found a high prevalence of polypharmacy and PIP (79.8% and 73.4%, respectively), especially above 75 years and in females. These findings confirmed that both are concerns in our elderly population and strengthen the need for active measures from doctors, such as drug reconciliation and timely deprescribing of PIP-associated drugs.

Facing these results, the authors will strive to remind medical teams of the adequate prescription in the elderly population of some of the classes primarily associated with PIP in this study, such as proton pump inhibitors. We expect to inspire other teams to study the prevalence of polypharmacy and PIP in their care settings to bring awareness to this growing issue.
